# Mycotoxin Biomarkers in Pigs—Current State of Knowledge and Analytics

**DOI:** 10.3390/toxins13080586

**Published:** 2021-08-23

**Authors:** Agnieszka Tkaczyk, Piotr Jedziniak

**Affiliations:** Department of Pharmacology and Toxicology, National Veterinary Research Institute, Partyzantow 57, 24-100 Pulawy, Poland; piotr.jedziniak@piwet.pulawy.pl

**Keywords:** mycotoxin biomarkers, pig health, multi-mycotoxin LC-MS/MS method

## Abstract

Farm animals are frequently exposed to mycotoxins, which have many adverse effects on their health and become a significant food safety issue. Pigs are highly exposed and particularly susceptible to mycotoxins, which can cause many adverse effects. For the above reasons, an appropriate diagnostic tool is needed to monitor pig’ exposure to mycotoxins. The most popular tool is feed analysis, which has some disadvantages, e.g., it does not include individual exposure. In recent years, the determination of biomarkers as a method to assess the exposure to mycotoxins by using concentrations of the parent compounds and/or metabolites in biological matrices is becoming more and more popular. This review provides a comprehensive overview of reported in vivo mycotoxin absorption, distribution, metabolism and excretion (ADME) and toxicokinetic studies on pigs. Biomarkers of exposure for aflatoxins, deoxynivalenol, ochratoxin A, fumonisins, T-2 toxin and zearalenone are described to select the most promising compound for analysis of porcine plasma, urine and faeces. Biomarkers occur in biological matrices at trace levels, so a very sensitive technique—tandem mass spectrometry—is commonly used for multiple biomarkers quantification. However, the sample preparation for multi-mycotoxin methods remains a challenge. Therefore, a summary of different biological samples preparation strategies is included in that paper.

## 1. Introduction

Mycotoxins are toxic fungal secondary metabolites present at agricultural commodities (mainly cereals) in all stages of production, processing and storage [1]. A few hundred mycotoxins with widely different chemical structures have been identified and reported so far [2]. The most common mycotoxins found in food and feed are: aflatoxins (AFLs); ochratoxin A (OTA); trichothecenes: deoxynivalenol (DON), T-2 toxin (T-2) and HT-2 toxin (HT-2); fumonisin B_1_ (FB_1_) and B_2_ (FB_2_) (fumonisins (FBs)) and zearalenone (ZEN). The World Health Organization has recognised these mycotoxins as a significant source of food-borne illnesses for animals and humans [3].

Mycotoxins are widespread contaminants of cereals, which are the most crucial feed ingredients. Pigs can be highly exposed to mycotoxins due to their cereal-based diet and are particularly susceptible to mycotoxins (paragraph 2). Ingestion of low mycotoxins levels may result in economic loss through clinically obscure changes in growth, production and immunosuppression than in an acute disease episode [4].

Different kinds of moulds produce hundreds of secondary metabolites, but only a few are regulated due to their scientifically proven adverse effects on human and animal health [5]. For this reason, the European Union set the maximum allowed concentration levels (for AFB_1_) or guidance values for some of the mycotoxins (DON, ZEN, OTA, T-2/HT-2 and FBs) in animal feed called “regulated mycotoxins”.

A classical approach to assess human and animal exposure to mycotoxins is based on a combination of contamination and consumption data [6]. Although, this approach has some disadvantages. First, among *Fusarium*–produced toxins, co-occurrence is frequently observed, and synergistic effects of *Fusarium* were reported in the past. Still, combinatory effects legislation does not take into account to date [7]. Next, a highly inhomogeneous distribution of a toxin in feedstuffs is observed, which led to irrelevant results [8].

Last but not least, another problem is the fact that feed analysis does not provide data about the individual exposure because of differences in food consumption and absorption, metabolism, and excretion (ADME) processes between the animals. Additionally, except for native mycotoxins, many so-called “modified mycotoxins” have been identified so far. Although there are no requirements to monitor them in feed, recently European Food Scientific Agency (EFSA) recommended their monitoring [9], and they are more and more detected in feed analysed for mycotoxins [9,10,11,12]. Toxicity data are missing for modified mycotoxins, but they could be hydrolysed into the parent compounds or released from the matrix during digestion. They may consequently contribute to unexpected high toxicity. This has been shown for 3- and 15-acetyl-deoxynivalenol (3- and 15-Ac-DON) and deoxynivalenol-3-glucoside (DON-3-Glc) in pigs and broiler chickens [13,14].

Due to the difficulties mentioned above, the exposure assessment of animals might include the analysis of toxin contamination in the diet and the analysis of samples of animal origin such as blood or urine to evaluate the individual toxin exposure. The presence of mycotoxin in biological matrices are called biomarkers of exposure and their analysis in biological matrices—biomonitoring.

This review provides a comprehensive overview of reported in vivo mycotoxin ADME and toxicokinetic studies on pigs concerning biomarkers of exposure for DON, ZEN, AFLs, T-2, OTA and FBs to select the most suitable biomarkers in porcine plasma, urine and faeces (paragraph 3).

Many mycotoxins are present in biological matrices at very low concentrations (below 0.1–10 ng/mL range). Therefore, selective and sensitive analytical methods are required making the high-performance liquid chromatography with tandem mass spectrometric detection (HPLC-MS/MS) the method of choice. There are no standardised methods to analyse mycotoxin biomarkers in biological matrices. Therefore, different biological samples preparation strategies for analysing multiple mycotoxins and most crucial, urine analysis challenges are summarised herein (paragraph 4).

## 2. Impact of Mycotoxins on Pig Health

The toxicological effects of regulated (in feed in Europe) mycotoxins on pigs have been reviewed in several papers [15,16], but also in the EFSA opinions (aflatoxin B_1_, 2004; deoxynivalenol, 2004; ochratoxin A, 2004, zearalenone, 2004, and fumonisins, 2005).

Recently, EFSA reported risks for animal health related to the presence of DON and ZEN and their modified forms in feed [17,18]. For these two mycotoxins, the EFSA Panel on Contaminants in the Food Chain (CONTAM) established no observed adverse effect levels (NOAELs) and lowest observed effect level (LOAEL) for pig (piglets and gilts). LOAELs are also established for OTA [19], FB_1_ [20] and T-2/HT-2 [21] (Table 1), but there is a lack of toxicological data to establish NOAELs.

### 2.1. Aflatoxins (AFs)

Among the mycotoxins, AFs are considered the most toxic [22]. These can cause various chronic or acute syndromes in pigs, depending on the level of consumption. Extreme effects can lead to death, but the most significant impact comes from weight loss and poor performance, reduced reproductive capability, changes in clinical biochemistry patterns, suppressed immune function, increased susceptibility to infectious diseases and increased mortality [23]. The liver is the primary target organ, with liver damage occurring when pigs are fed aflatoxin B_1_ [5].

Aflatoxins are hepatotoxic and carcinogenic; they also display immunotoxic properties in pigs [24]. Aflatoxins could impair both the cellular and humoral immune systems. The general mechanism of the immunosuppressive effects of AFB_1_ is associated with the impairment of the synthesis of proteins [25]. Low doses (140 and 280 ppb) of AFs depress growth and alter many aspects of humoral and cellular immunity in pigs [23].

### 2.2. Deoxynivalenol (DON)

Reduced feed intake and reduced body weight gain were the most relevant chronic adverse effects of DON in pigs. However, DON may cause several other adverse effects in pigs, including lesions in the stomach’s oesophageal region, the liver, the lungs and the kidneys, and changes in different clinical chemistry parameters (plasma nutrients and plasma enzyme activities) [26]. The feeding of contaminated feed containing DON and decreasing food intake can influence the immune responses in pigs. Still, the types and the sizes of those responses could not be associated with relevant adverse immunological effects in pigs [24].

The CONTAM panel identified for the acute adverse health effects a lowest-observed-adverse-effect level (LOAEL) of 2.8 mg DON/kg feed for vomiting. For reduced feed intake and reduced weight gain reduction, identified as the acute, chronic adverse health effects of DON in pigs, wide ranges of NOAEL and LOAEL values were reported from an overall NOAEL of 0.7 mg DON/kg feed was identified by the CONTAM panel [17]. 

### 2.3. Fumonisin B_1_ (FB_1_) and Fumonisin B_2_ (FB_2_)

Consumption of high doses of fumonisins over short periods by swine can induce porcine pulmonary oedema (fluid accumulation in the lungs, probably due to left-side heart failure, usually lethal) [27]. It can also cause other diverse pathological lesions such as cardiovascular lesions or pancreatic necrosis [28] and hepatic intoxication [29].

The mechanism of action of fumonisins involves inhibition of the enzyme ceramide synthase, a key enzyme in the biosynthesis of sphingolipids [30]. Considering the Sa:So (sphinganine:sphingosine) ratio as the most sensitive parameter in the assessment of adverse effect exerted by fumonisins, the LOAEL was found to occur when pigs were exposed to feed containing 5 mg of fumonisins per kg feed (which is corresponding to approximately 0.2 mg/kg body weight (b.w.)/day) [20]. Lung lesions typical for fumonisins toxicity in pigs were observed at a dose of 0.4 mg/kg b.w./day [31,32].

A recent study with pigs fed with FBs-contaminated diets at doses below the EU regulatory limit shows that even a dose of 3.7 mg/kg feed, i.e., below the regulatory limit of 5 mg FB_1_/kg feed, has harmful effects on the heart and intestine of piglets. This study established a LOAEL of 3.7 mg/kg corresponding to 148 µg/kg b.w. per day [33].

### 2.4. Ochratoxin A (OTA)

The pig is much more sensitive to the adverse effects of ochratoxin A than most laboratory animal species except dogs [34]. OTA can cause a variety of chronic or acute syndromes depending on the level of consumption. OTA is known primarily for its nephrotoxicity. In pigs, it causes kidney lesions, and this pathology is called MPN (mycotoxic porcine nephropathy) [35]. Progressive nephropathy is seen in pigs at dietary concentrations of 1 mg/kg (equivalent to 40 µg/kg b.w.) [36]. OTA residues have been relatively often found in the kidney of pigs [37,38]. Zootechnical impacts have also been observed: poor weight gain, feed intake and feed efficiency. Low levels of OTA can induce several clinical symptoms, such as polydipsia and polyuria, while higher levels can provoke vomiting, anorexia, serious diarrhoeas, and even the death of pigs [39].

No data are available for establishing a NOEL, but based on effects on renal (diagnostic) enzyme levels and kidney function, the dietary concentration of 0.2 mg/kg (equivalent to 8 µg/kg b.w.) is considered to be the LOAEL [19].

### 2.5. T-2 Toxin (T-2)

Chronic exposure to T-2 toxin reduces feed intake and body weight gain in pigs. Similarly, as with other trichothecenes, T-2 is an inhibitor of protein synthesis. It is also hepatotoxic and immunotoxic with harmful effects on the cell-mediated and humoral acquired responses [40]. Acute exposure to T-2 toxin induces oxidative stress in the liver and lipid peroxidation. It has recently been demonstrated that sub-clinical doses of T-2 toxin have harmful effects on liver metabolising enzymes [39].

For pigs, the most sensitive endpoints are immunological or haematological effects from 29 µg/kg b.w. per day (equivalent to 500 µg T-2 toxin/kg feed). Based on the available data, 29 µg T-2 toxin/kg b.w. per day could be considered as a LOAEL. So far, no NOAEL is identified for pigs. The CONTAM panel concluded that a reduction in specific antibody response in pigs is the critical effect for human risk assessment [21].

### 2.6. Zearalenone (ZEN)

ZEN can cause different harmful health effects in pigs. Mainly reported alterations of fertility and reproduction [26]. This effect results from the capacity of ZEN to bind the oestrogen receptors [41]. Prepubertal female piglets are susceptible to ZEN exposure. The main reason for ZEN sensitivity is that swine convert ZEN to the more estrogenically active metabolite α-zearalenol (α-ZEL) [42].

ZEN is also a potential hepatotoxin when administered through the oral route because it altered several important hepatic cellular immune response [43,44]. It has also been shown that spleen and blood may also be target tissues in weanling piglets fed ZEN contaminated diet, with different effects on oxidative stress and inflammation [45].

In addition, low feeding levels of ZEN reduced nutrient digestibility, increased oxidative stress and negatively affected pigs’ growth [46].

There is an extensive data set for ZEN toxicokinetics in pigs, including piglets, gilts, and sows. In all porcine categories, the production of α-ZEL largely outweighed that of β-ZEL and other reductive metabolites, which were recovered, along with ZEN in blood and urine mostly in their glucuronidated form [18].

Estrogenic effects such as increased uterus weight and reddened and swollen vulva were observed in pigs given 17.6 µg ZEN/kg b.w. (LOAEL). The prolonged cycling of sexually mature female pigs was reported for cycling sows from 200 µg/kg b.w. per day (LOAEL), with no effect at 40 µg/kg b.w. per day, which is the lowest reported NOAEL for mature female pigs [18]. Pigs and sheep were found to be the most susceptible species. Therefore, in the human ZEN risk assessment from EFSA in 2011, the human tolerable daily intake (TDI)—0.25 µg/kg b.w.—was based on a NOEL of 10 µg/kg b.w. per day for oestrogenic effects in pigs [18].

### 2.7. Modified Mycotoxins

For modified forms, no reference points could be established for any animal species. To assess occurrence in feed and exposure of animals, the CONTAM Panel added equal factors for masked mycotoxins in feed as in food; 100%, 30%, 10% and 60% to the levels of the parent compounds to account for the modified forms of zearalenone, nivalenol, the sum of T-2 and HT-2 toxin and fumonisins, respectively. In EFSA opinion, after including modified forms, re-assessments of animal health effects of zearalenone and fumonisins are needed to set NOAELs/LOAELs for these compounds. Still, toxicological data on modified mycotoxins are missing [9].

## 3. In Vivo Mycotoxin Absorption, Distribution, Metabolism and Excretion (ADME) and Toxicokinetic Studies on Pigs

Recently, several in vivo studies were carried out in pigs. In excretion studies after oral administration (PO) of mycotoxins, biomarkers were detected in urine and/or faeces and/or serum. In toxicokinetic studies after oral or/and intravenous (IV) administration of mycotoxins, biomarkers were detected in plasma/serum. Only non-invasive matrices—urine, faeces and blood—are summarised in this review.

The collection of biological matrices from pigs is much difficult compared to a human. Therefore, a limited study was carried out with pigs.

Usually, after one week of acclimatisation period, in most studies, DON, ZEN and their metabolites or combination of these mycotoxins were administrated mainly in small pig groups (4 pigs per group) for 2.5–37 days. Although, there were two studies with significant numbers of pigs (more than a hundred female piglets) divided into different feedings groups, which resulted in more than 20 pigs per group [47,48]. Usually, pigs were housed individually in metabolic cages [49,50,51,52], rarely in pens [53]. Feed for experiments was prepared naturally contaminated with oats, wheat or maize or pure toxin was added into feed. Although, in most studies, an aqueous stock solution of mycotoxins was administrated in pigs.

Recently, there were five dose–response studies in pigs with four/five ZEN (Table 2) and DON (Table 3) feeding groups [47,48,49,54,55]. ZEN and DON biomarkers were analysed in urine in each of these studies and plasma only in three studies [47,48,54]. LC-MS/MS analyses were carried out in every study except for two HPLC methods [48,54]. ZEN and its metabolites were administrated in two studies, and biomarkers were found in urine, faces and serum [53,56]. DON and its metabolites were administrated in three studies, and biomarkers were found in urine, faces and serum [13,57,58].

The sampling time for all matrices lasted from the start of exposure until 2 days (48 h) after the last ration of contaminated feed was given to the pigs. The prolonged experiment in pigs is missing. Urine was mainly collected before slaughtering (Table 2 and Table 3)—only in one of the dose–response studies was collected during 24 h [49]. In another study, urine was collected twice a day—in the morning after 8h fast and in the afternoon—after 7 h of ab libitum access to feed [55].

Zeranol (α-zearalanol) has been widely adopted as a growth stimulant, whereby its application has been banned in the European Union since 1985 (Council Directive 85/649/EEC). Therefore, urine was also collected from pigs to study the presence of this hormone and its metabolites (β-zearalanol, α-zearlenol and β-zearlenol) [59].

### 3.1. ZEN Administration

#### 3.1.1. ZEN Urinary Biomarkers

The dose of ZEN for the piglets and gilts in experimental diets was between 4 and 290 μg ZEN per kg feed (Table 2), whereas the guidance value for feed is 100 µg/kg feed. A much higher dose of ZEN was administered to male pigs—350–2360 µg/kg [49], compared to the guidance values for feed for sows—250 µg/kg.

The time of urine sampling (a.m. vs. p.m.) did not influence urinary ZEN concentration. The long half-live of ZEN explains why urinary ZEN is more highly correlated to the mean ZEN intake during the 3 days compared to ZEN intake 1 day before urine was collected [55].

Glucuronides of zearalenone (ZEN-GlcAc) and/or its metabolites (α-ZEL-GlcAc) were determined in urine directly only in three studies with pigs—with synthesised standards (ZEN-14-GlcAc) [55,56] or with HR-MS [60]. Mainly, they were determined indirectly—after enzymatic hydrolysis (sum of unconjugated plus conjugated metabolites of ZEN) due to lack of commercially available standards.

It has been shown that zearalenone is predominantly excreted in urine as glucuronides of zearalenone and α-zearalenol (α-ZEL). The presence of low concentrations of β-zearalenol (β-ZEL), compared to α-ZEL, in the urine of pigs fed with diet contaminated with ZEN was often reported in recent studies. In particular, for urines containing both β-ZEL and α-ZEL, the ratio α-ZEL/β-ZEL ranged between 17.5–73.7 [49,54]. The concentration of ZEN and its metabolites: α-ZEL, β-ZEL and zearalanone (ZAN) increased in pig urine samples with increasing median concentrations, whereas α-and β-ZAL were only detected sporadically [47].

**Figure 1:**
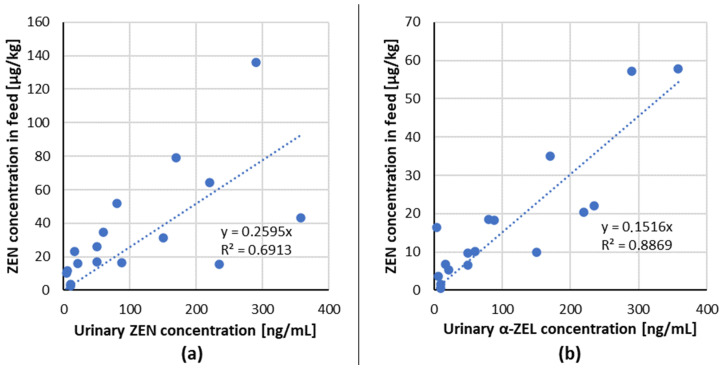
Correlation of ingested mycotoxin dose compared to the relevant urinary biomarker(s) excreted in 24 h post-dose for each mycotoxin/biomarker couple from studies in pigs (data from Table 2) (**a**) ZEN/ZEN, (**b**) ZEN/α-ZEL.

The linear regression analysis of ingested mycotoxin dose compared to the relevant urinary biomarker(s) for ZEN/biomarker (ZEN, α-ZEL) couple from dose–response studies with pigs (Table 2) are shown in Figure 1.

A positive linear dose–response relationship for the tested combinations of mycotoxin/biomarker from studies with pigs has been demonstrated, indicating that ZEN and its metabolites: α-,β-ZEL in urine are suitable biomarkers of exposure in pigs.

#### 3.1.2. Faecal Biomarkers

Following oral administration of ZEN in lyophilised faeces, ZEN and α-ZEL were detected. The recovery of the applied toxin in faeces (24–48 h after application) ranged from 10% and 18% [56].

Biomarkers of ZEN were also detected in another study in pig faeces [47]. The concentration-time profiles of ZEN and its phase I metabolites: α-,β-ZEL, and ZAN in faeces excreted via faeces showed maximum levels from the first 12 h after exposure onwards. The highest amounts were excreted during 12–24 h. These results consist with other studies—ZEN in pig dried faeces was selected with its largest amount being excreted after 24 h [60].

These two above described studies showed that ZEN and its metabolites: α-,β-ZEL, and ZAN in faeces can also be suitable biomarkers of exposure in pigs, but more studies are needed to prove this thesis.

#### 3.1.3. Serum Biomarkers

Pig serum samples were collected in three dose–response studies with pigs. All serum samples were analysed after enzymatic hydrolysis (as a sum of the conjugated and unconjugated form). Neither ZEN nor its metabolites were detected in the serum of the piglets when serum samples were analysed with HPLC-FLD method. This is probably due to relatively high LOD for ZEN (1 ng/g for sample weight 5 g) [48]. In two other studies, serum samples were analysed with LC-MS/MS method, resulting in much lower LOD (for ZEN—0.03 ng/mL). ZEN concentration in serum increased slightly for the groups receiving 0.01, 0.05, 0.08, 0.17 and 0.29 mg ZEN/kg diet when the maximum concentrations were considered. The metabolite α-ZEL detected only sporadically in serum of Groups 4 and 5-diet with ZEN concentrations above the guidance value (0.1 mg ZEN/kg diet)—at concentration levels up to 1.6 ng/mL—the individual ratios of ZEN-to-α-ZEL ratios were approximately 1/3 [47]. In the second dose–response study, only small amounts of ZEN were quantified in groups 3–5 (0.08–0.29 mg ZEN/kg) with median ranged between 0 and 0.35 ng/mL, whereas α-ZEL was detectable in groups 3–5 but could only be quantified in group 5 with a median of 0.44 ng/mL. Neither ZAN, α-ZAL, β-ZAL, nor β-ZEL could be detected [61].

The analysis of pig serum samples using the LC-HRMS instrument showed that ZEN-GlcA more suitable biomarker for exposure than ZEN. The ZEN-GlcA peak area is much higher than those of the respective parent component ZEN in another study with pigs (after oral administration of 3 mg/kg ZEN—much higher than the guidance value) [60].

The analysis of ZEN and its metabolites in serum showed that they are not suitable as biomarkers for dietary ZEN exposure/intoxication. These concentrations either were below the LOQ or could only be detected in the feedings groups administered ZEN in a much higher dose than the guidance value.

Recently, pig serum/plasma samples were analysed to study the metabolism and/or pharmacokinetics of ZEN in pigs in three studies [47,61,62]. Analysing the time-plasma concentration profiles (after administration of mycotoxins), we can observe the time (Tmax) when the maximal concentration of toxin (Cmax) was achieved. This time should be considered as sampling time. Therefore, biomarkers in dose–response studies were found at very low concentrations when samples were taken before or after Tmax.

The Tmax after oral administration of 1.0 mg/kg b.w. of ZEN was for: ZEN 0.25–2 h, α-ZEL 0.25–4 h, β-ZEL 0.5–9 h. The fraction of total α- and β-ZELs present in plasma in unconjugated forms was small and similar to those seen for ZEN. The mean Cmax values for α-ZEL exceeded that for β-ZEL, and both were lower than that for ZEN [62].

#### 3.1.4. Administration of ZEN and Its Modified Forms

Metabolism of zearalenone and its major modified forms (ZEN-14-sulfate (ZEN-14-S), ZEN-14-O-β-glucoside (ZEN-14-Glc) and ZEN-16-O-β-glucoside (ZEN-16-Glc)) in pig urine and faeces was studied once [56].

Following oral administration of modified forms of ZEN, the analyte itself was neither detected in urine nor faeces samples. ZEN-14-Glc, ZEN-16-Glc, and ZEN-14-S were readily hydrolysed to ZEN and converted to other still-unknown metabolites in the gastrointestinal tract of pigs.

Recently, absolute oral bioavailability, biotransformation, and toxicokinetics of ZEN and their metabolites: α-ZEL, β-ZEL, ZEN-14-Glc, and ZEN-14-S in pigs was investigated in one study [53]. After oral administration, plasma concentrations of ZEN, its modified forms, and its phase I metabolites were too often below the LOQ to construct reliable plasma-concentration-time profiles. Results demonstrate complete hydrolysis of ZEN-14-Glc and ZEN-14-S to ZEN and high oral bioavailability for all administered compounds, with further extensive first-pass glucuronidation. In contrast to ZEN and its phase I metabolites, the GlcA metabolites of ZEN, α-ZEL, and β-ZEL could be detected after oral administration, demonstrating systemic exposure to all orally administered ZEN forms and demonstrating the potential of these phase II metabolites as biomarkers for ZEN exposure in pigs.

### 3.2. Deoxynivalenol Administration

#### 3.2.1. DON Urinary Biomarkers

The metabolism of DON in swine explain why this species is more susceptible to DON than other animals. Indeed, DON is rapidly and efficiently absorbed, extensively distributed in tissues and body fluids, and poorly metabolised in pigs [63].

The pigs are fed the experimental diet ingested between 30–4520 µg DON/kg feed (Table 3)—the highest dose was much higher than the guidance value for pig feed—900 µg DON/kg feed.

The time of urine sampling (a.m. vs. p.m.) influenced urinary DON concentration. The correlation between DON intake and urinary DON/creatinine ratio in the urine collected in the afternoon was higher for the intake during the preceding 7 h (r = 0.88) than that for the intake during the previous day (r = 0.76) [55].

Similar to ZEN, glucuronides of deoxynivalenol (DON-GlcAc) were determined directly only in three studies with pigs—with HR-MS [64] or with synthesised standards (DON-3-GlcAc/DON-15-GlcAc) [55,57].

In all dose–response studies, DON and DOM-1 were detected in all feeding groups’ urine samples in increasing concentrations corresponding to increasing DON concentration in the diets and most cases with significant differences between the groups of one experimental day.

DON showed the most important urinary biomarker (20–30% of the administrated dose was excreted as DON). The maximum concentrations were achieved after 4–8 h for DON [57,64]. The presence of low concentrations of DOM-1 was found in all dose–response studies. DON-3-GlcAc and DON-15-GlcAc in urine at low concentration after DON administration was reported in two DON metabolism studies [57,64].

**Figure 2:**
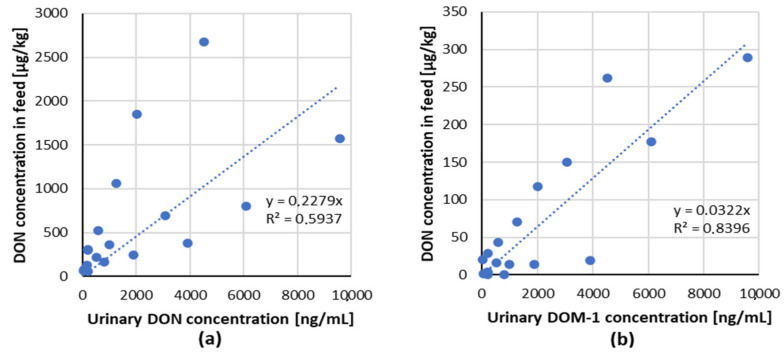
Correlation of ingested mycotoxin dose compared to the relevant urinary biomarker(s) for each mycotoxin/biomarker couple from studies in pigs (data from Table 3). (**a**) DON/DON, (**b**) DON/DOM-1.

The linear regression analysis of ingested mycotoxin dose compared to the relevant urinary biomarker(s) for DON/biomarker (DON and DOM-1) couple from dose–response studies with pigs are shown in Figure 2 [47,49].

A positive lineal dose–response relationship for the tested combinations of mycotoxin/biomarker from two studies with piglets has been demonstrated, indicating that DON and its metabolite DOM-1 in urine are suitable biomarkers of exposure in pigs.

#### 3.2.2. DON Serum Biomarkers

Pig serum samples were collected in three dose–response studies with pigs. All serum samples were analysed after enzymatic hydrolysis, and the DON was expressed as a sum of the conjugated and unconjugated form). Only small concentrations of DON and DOM-1 were found in serum. The correlation coefficients (>0.8) indicate that the DON and the sum of DON and DOM-1 concentration in serum correlated well with the dietary DON concentration. The individual ratio of DON/DOM-1 in serum was approximately 5/1 [47,61]. The effect of incubation of serum samples with beta-glucuronidase before HPLC analysis revealed a significant increase in concentration for each individual pig sample. The degree of conjugation of DON in serum was approximately 33% (19–45%), whereas that of de-epoxy-DON could not be evaluated since its concentrations in enzymatically untreated serum samples were all lower than the detection limits [54].

**Figure 3:**
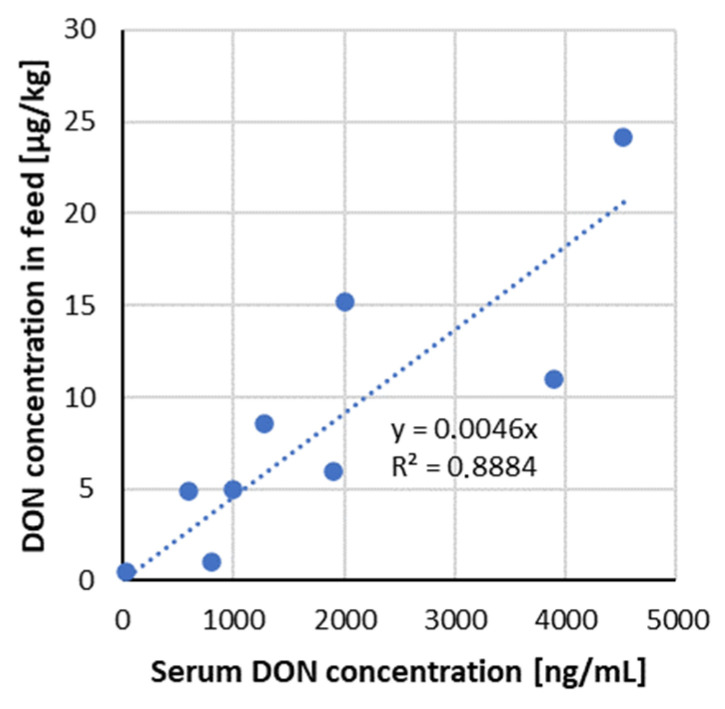
Correlation of ingested mycotoxin dose compared to the serum DON concentration—data from recent studies in pigs (Table 3).

The analysis of DON in serum showed that it is suitable as a biomarker for dietary DON exposure/intoxication since its concentration increased linearly, corresponding to increasing DON concentration in the diets (Figure 3). DOM-1 was found in serum samples only in groups, which received higher DON doses (>2000 µg/kg feed).

#### 3.2.3. DON Faecal Biomarkers

The elimination of DON via faeces accounted for only 1.8 ± 1.6% [57]. DON and DOM-1 were also detected in another study in pig faeces but at levels below LOQ (5 ng/g) [60].

DON and DOM-1 detected in all studies with pigs in faeces were below the limit of quantification and could thus not be considered biomarkers.

### 3.3. Administration of DON and its Modified Forms

The three experiments studied levels of deoxynivalenol and its major modified forms (DON-3-glucoside, 3-acetyl-deoxynivalenol) in pig urine, faeces and plasma [13,57,58].

Following oral administration of modified forms of DON, the analyte itself was neither detected in urine nor faeces samples. No traces of 3-AcDON or its de-epoxide metabolite were found in plasma, urine or faeces.

The amount of DON found in faeces only accounts for 2% of the total amount of 3-AcDON given to the pigs [58]. After oral DON-3G administration, in faeces, just trace amounts of metabolites were found. DOM-1 was found in faeces samples 8–24 h after dosing.

Recently, pig serum/plasma samples were analysed to study DON’s metabolism and/or pharmacokinetics in pigs in three studies.

The Tmax after oral administration of 36 µg/kg b.w. of DON, 41 µg/kg b.w. of 3-AcDON and 15-AcDON was for DON 109 ± 41 min, 3-AcDON 101 ± 45 min, 15-AcDON 78 ± 39 min. The administered dose of both AcDONs is completely absorbed, and both show a complete presystemic hydrolysis. Therefore, both AcDONs can be regarded as toxic as DON in pigs concerning systemic toxicity [13].

Five castrated pigs were fed twice daily for 3 days 2.5 mg 3-AcDON/kg feed. No 3-AcDON could be detected in plasma. The only detected metabolite in plasma was DON as such. After incubation of the plasma samples with β-glucuronidase, an increase of 72% DON was seen, indicating the presence of DON-glucuronide. The maximum concentration of DON in plasma was reached after 3 h and decreased rapidly after that. Only low concentrations close to the detection limit were found in plasma 8 h after the feeding [58].

The analysis of pig serum samples using the LC-HRMS instrument also showed that DON-GlcA is a better biomarker for exposure as its observed peak area is much higher than those of the respective parent component DON in another study with pigs (after oral administration of 36 µg/kg b.w. ZEN—the guidance value) [64].

### 3.4. Other Mycotoxins

As shown in Table 4, there were a few in vivo studies on pigs with the administration of other mycotoxins (except DON and ZEN). OTA was frequently administrated to pigs for a longer time (up to 180 days). In most in vivo studies, besides urine and plasma, pig’s tissues were collected because feeding on OTA-contaminated feed leads to OTA accumulation in edible pig tissues used in the human diet.

#### 3.4.1. AFB_1_ Administration

Two animal studies with pigs reported the urinary excretion of AFB_1_ after oral administration of the toxin. Mean percentage of ingested AFB_1_ (0.16–1.28 µg/kg b.w.) excreted as AFM_1_ biomarker in the 24 h post-dose urine was 2.5% [49]. The urinary levels of AFB_1_ fed AFB_1_-contaminated diets (containing 127, 227 and 327 µg AFB_1_/kg feed) were determined using LC-MS/MS. Biomarkers of AFB_1_ in the pig urine, AFM_1_, AFB_1_, and AFB_2_ were detected at 4–32, 1–35, and 0.4–0.9 ng/mL, respectively. The urine concentrations of AFB_1_ and AFM_1_, and the sum did not differ significantly between the diet levels. Between 20 and 48% of the consumed AFB_1_ dose was excreted into urine as AFB_1_ and AFM_1_. The proportion of converted to AFM_1_ was on average 22% of the dose. The results indicate that the metabolism and excretion of AFB_1_ change with increasing feed concentrations. Low amounts of AFB_2_ were also found in the pig urine. The concentration was low and similar on all diets. This may have resulted from the excretion of the natural contamination of AFB_2_ in the feed used for the trial. A conversion from AFB_1_ cannot, however, be excluded [50].

#### 3.4.2. OTA Administration

Although OTA was often administrated to pigs, urine was collected after 24 h only once [49]. The mean percentage of ingested OTA (0.16–1.32 µg/kg b.w.) excreted as a biomarker in the 24 h post-dose urine was 2.6%. Linear regression analysis of ingested OTA dose compared to OTA excreted in 24 h post-dose showed R^2^ of 0.68 [49]. In most studies with pigs ingested OTA, biological matrices (plasma/serum or urine) were collected once before slaughtering (Table 4). In two studies, in which pigs were fed with naturally contaminated feed [67,68], higher OTA serum/plasma levels were found compared to studies in which pigs were fed OTA fortified feed (Table 4). Recently, ochratoxin α (OTα) was quantified for the first in pig urine—at two times lower concentration than OTA [71], indicating an important urinary OTA biomarker and should be included in future OTA biomonitoring. Several studies have shown that OTA has an extremely high affinity for serum albumin and other macromolecules in the blood. Therefore the elimination half-life of OTA in pigs is very long—72–120 h [74]. That fact can be beneficial for OTA serum biomonitoring.

#### 3.4.3. FB_1_ Administration

A dose–response study after FB_1_ administration to pigs was reported only twice. The mean percentage of ingested FB_1_ (3.7–150 µg/kg b.w.) excreted as a biomarker in the 24 h post-dose urine was 2.6%. Linear dose–response correlation coefficients was 0.76 [49].

The levels of fumonisin B_1_ (FB_1_) residues in plasma, urine, faeces and hair fed FB_1_- contaminated diets (containing 3.1, 6.1 or 9.0 mg FB_1_/kg) were determined using LC-MS/MS. The levels of FB_1_ in plasma, urine, faeces and pooled hair samples varied from 0.15–1.08 ng/mL, 16.09–75.01 ng/mL, 1.87–13.89 µg/g and 2.08–8.09 ng/g, respectively. Significant correlations (R^2^ 0.808–0.885) were found between FB1 intake and plasma FB1 on days 7, 14, 21 and 28. However, urinary FB_1_ correlated with FB_1_ intake only on days 7 and 14 (R^2^ 0.561–0.572). A significant correlation (R^2^ 0.509) was also found for the first time between FB_1_ in hair samples and FB_1_ intake.

Plasma and urinary FB_1_ are promising biomarkers of early exposure of pigs to low dietary FB_1_ levels, although plasma is recommended to assess prolonged exposure (>14 days). The possibility to evaluate hair as a biomarker of fumonisin exposure was established, although further studies are needed to provide physiologically-based toxicokinetics of residual FB_1_ in the pig hair [73].

Toxicokinetics and the toxicological effects of culture material containing FB_1_ were studied in male weaned piglets. The animals received a single oral dose of 5 mg FB_1_/kg b.w. The highest concentration of FB_1_ in plasma was observed after 2 h, with a mean concentration of 282 µg/mL. Only 0.93% of the total FB_1_ was detected in urine between 75 min and 41 h after administration. The highest mean concentration (561 µg/mL) was observed during the interval after 8 at 24 h. Approximately 76.5% of FB_1_ was detected in faeces eliminated between 8 and 84 h after administration, with the highest levels observed between 8 and 24 h [72].

The distribution and elimination of fumonisins after oral administration of 50 mg FB_1_, 20 mg FB_2_ and 5 mg FB_3_ per animal per day for 22 days were studied in weaned barrows. At the end of the trial, the serum, urine and faeces samples were collected, and LC-MS determined their content of fumonisins (FB_1_, FB_2_). Of the total quantity of FB_1_, the 13% taken up during 5 days was excreted unchanged with the faeces and urine. On average, in the urine and faeces, FB_1_ was detected in nine- and 14-fold quantities compared with FB_2_ [52].

#### 3.4.4. T-2 Administration

Toxicokinetics and excretion studies of T-2 toxin and its major metabolites after intravenous (i.v.) administration in pigs was carried out only once [51]. Six pigs received T-2 toxin intravenously as a single dose at 1 mg/kg b.w. to study the toxicokinetics of T-2 and its major metabolites HT-2 and T-2 triol. The plasma concentrations of T-2 toxin, HT-2 toxin, and T-2 triol after i.v. administration were all detected until after 3 h, which indicated that the pigs absorbed and metabolised T-2 toxin. The mean T-2 Cmax were much higher than those of HT-2 toxin and T-2 triol. Seven pigs received i.v. T-2 toxin as a single dose at 0.5 mg/kg b.w. to study the excretion of T-2, HT-2, and T-2 triol in urine and faeces. The excretion data of T-2 toxin, HT-2 toxin, and T- 2 triol in urine indicated that merely <7% (2.41%, 3.63%, and 6.32%, respectively) of the dose administered was excreted, and the excretion peak time of metabolites HT-2 toxin and T-2 triol was 12–24 h. T-2 toxin and T-2 triol were not observed in the faeces; only a small amount of HT-2 toxin was excreted (0.25% of the administered dose).

### 3.5. The Most Suitable Biomarkers Found in Different Biological Matrices

The aim of the summary of above mentioned in vivo studies was to select relevant biomarkers of exposure in other pig biological matrices: urine, faeces and plasma/serum (summarise in Table 5).

Additionally, the good dose–response correlations make the urinary multi-biomarker approach an excellent tool to assess in vivo the efficacy of mycotoxin detoxifying agents in reducing the bioavailability of mixtures of mycotoxins [49]. This approach is suitable for DON and DOM-1 in urine, DON in serum, ZEN and α-ZEL in urine. More dose–response studies with administration of other mycotoxins (except DON and ZEN) are needed to select suitable biomarkers in different biological matrices and find the eventual correlation of ingested mycotoxin to the excreted biomarker.

Adequate sampling time plays a significant role in in vivo experiments, as every mycotoxin has another excretion profile in different biological matrices. The 24 h urine collection is preferred in multi-mycotoxin studies because, for most analytes, very little is known about the kinetic profile of mycotoxin biomarkers. Toxicokinetic studies should be taken into account by serum sampling to know the maximum concentration of mycotoxins, which were rapidly excreted from blood (such as DON and ZEN) (Table 5).

## 4. Methods for Mycotoxin Biomarkers Analysis

As shown in Section 3, mycotoxin biomarkers occur in biological matrices at trace levels. Therefore, multi-analyte methods based on liquid chromatography-tandem mass spectrometry (LC-MS/MS) are commonly used to assess mycotoxin exposure. A limited number of LC/MS/MS methods are developed to determine biomarkers in pigs urine (Table S1) compared to human urine (much more developed methods). The LC-MS/MS methods for mycotoxin biomarkers determination in human urine were summarised and compared by Warth et al. in 2013 [75].

As shown in Table 5, DON and ZEN are excreted in pig biological matrices in the form of free and conjugated forms as glucuronides. Generally, mycotoxin glucuronides cannot be quantified directly because of the lack of standard materials [76]. Therefore, in most studies, urine and serum samples were digested with β-glucuronidase to break down the conjugated forms and obtain more accurate exposure results.

The most frequently used column for analysing multiple mycotoxins (also very polar) is the C18 column [64,77]. Mobile phases usually consisted of water and acetonitrile [51,56] or methanol [64,78]. Ammonium acetate was commonly used as a mobile phase additive and had significantly higher signal intensity for the majority of mycotoxins than ammonium formate (Table S1).

From an analytical perspective, urine is a challenging matrix due to vast differences in composition and concentrations between individuals, which may depend on sex, age, health status, metabolism and predominantly diet [75]. Therefore, creatinine concentration in urine can be taken to measure the dilution of the urine [79]. The range of creatinine content can differ among the species, from lower values in humans (0.28–2.59 mg/mL) [79] and higher for pigs (0.07–10.77 mg/mL) [80]. In the case of urinary mycotoxin biomarkers in pigs, there are only three studies in which standardisation for different dilutions of urine samples was used [55,56,81].

Because of the chemical diversity of multiple mycotoxin biomarkers, optimisation of the sample preparation procedure is the most challenging step for method development. Different sample preparation strategies for pig urine, serum and faeces samples with LC-MS/MS analysis are summarised in Table S1.

### 4.1. Urine Sample Preparation

Regarding the articles analysed the most LC-MS/MS method are developed for major *Fusarium* mycotoxins that correspond with those legislated (in feed) mycotoxins (DON, ZEN, T-2, HT-2, OTA and FBs) or combinations of these [47,55,57,82]. However, only a few are focused on other mycotoxins: neosolaniol (NEO), lysergol (LYS), methylergonovine (MET), roquefortine C (RC), wortmannin (WOR), verruculogen (VER) [78], emerging mycotoxins (beauvericin (BEA), enniatins (ENNs)) and Alternaria toxins (tenuazonic acid (TeA), alternariol (AOH), alternariol monomethyl ether (AME)) [64].

A wide variety of sample preparation protocols has been reported in the literature for multi-mycotoxin methods in pig urine: liquid-liquid extraction (LLE), solid-phase extraction (SPE), immunoaffinity columns (IAC) and dilute-and-shoot approach (D–S).

The first published LC-MS/MS method described for the determination of ZEN and its metabolites (α-ZEL/ZAL and β-ZEL/ZAL) in pig urine was developed by Jodlbauer et al. and applied RP-18 SPE columns after enzymatic hydrolysis with β-glucuronidase from *Helix pomatia* [59]. Limits of detections (LODs) and limits of quantifications (LOQs) ranged between 0.1–0.5 ng/mL and 0.5–1.0 ng/mL, respectively. Satisfactory recovery (94–105%) was achieved in this study. The extraction on SPE columns (Oasis™ HLB) after enzymatic hydrolysis with β-glucuronidase from *Helix pomatia* was also performed for ZEN and its metabolites and additionally, DON and DOM-1 with satisfactory recovery (76–118%) [47]. Triple-quadrupole mass analyser with better sensitivity was used. That could explain lower LODs (0.03–0.16 ng/mL) and LOQs (0.1–0.52 ng/mL) achieved for ZEN and its metabolites as in the first study. In this study, low LODs/LOQs were achieved for DON (0.11/0.38ng/mL) and DOM-1 (0.04/0.15 ng/mL). Matrix effects were not determined in any study with SPE columns application.

To achieve sufficient sensitivity and selectivity, DON, DOM-1, AFM_1_, OTA, FB_1_, ZEN, α- and β-ZEL were concentrated after enzymatic hydrolysis with β-glucuronidase from *Helix pomatia* using Myco6in1 immunoaffinity column (IAC) (Vicam) and an OASIS HLB solid-phase extraction (SPE) column (Waters) connected in tandem [81]. The eluates were pooled, evaporated under a stream of nitrogen, and resolved in a mixture of methanol and water. Advanced clean-up procedure resulted in lower LODs/LOQs for ZEN (0.02/0.07 ng/mL), α-ZEL (0.04/0.13 ng/mL) and β-ZEL (0.04/0.15 ng/mL) and similar for DON (0.18/0.61 ng/mL) and DOM-1 (0.36/1.21 ng/mL), of this method compared with that applied SPE columns. Additionally, very low LODs/LOQs were achieved for AFM_1_ (0.01/0.03 ng/mL), OTA (0.006/0.02 ng/mL) and FB_1_ (0.02/0.06 ng/mL). Recovery ranged from 64% for FB_1_ to 100% for α-ZEL. The matrix effect was not assessed in this study. It was the only application IAC for pig urine samples. In contrast to human mycotoxin biomonitoring studies, IAC was often applied [83,84,85].

The dilute-and-shoot (D–S) approach was applied in three methods for the determination of DON, ZEN and their metabolites in pig urine samples [55,56,57]—very rarely compared to human urine [86,87]. DON, D3G, DOM-1 as well as glucuronides: DON-15-GlcAc and DON-3-GlcAc were analysed after dilution of urine (1:9) with the mixture of MeOH/water (20/80, *v*/*v*) [57]. In this study, glucuronides of DON (quantification of DON-15-GlcA was performed using DON-3-GlcA standards) were for the first time directly quantified in pig urine samples with LOD/LOQ for DON-3-GlcAc 9/37.3 ng/mL. The D–S approach resulted in about ten times higher LODs/LOQs for DON (0.9/8 ng/mL) and four times higher for DOM-1 (1.4/3.7 ng/mL) compared to SPE and IAC columns. LOD/LOQ for D3G was 1.3/2 ng/mL. The apparent recovery ranged from 56% for DON to 114% for DON-3-GlcAc. Matrix effects were assessed in this study. Significant signal suppression was found for DON (56%), D3G (62%), DOM-1 (72%) and signal enhancement for DON-3-GlcAc (122%).

The second application of D–S for sample preparation of pig urine samples was to analyse DON and its metabolites: DON-3-GlcAc, DON-15-GlcAc, DON-3-sulphate, DOM-1 as well as ZEN and for the first time its metabolites: ZEN-14-GlcAc, α-ZEL and α-ZEL- 14-GlcAc [55]. Before analysis, all urine samples were diluted to the same creatinine concentration (0.2 mM). Analyte concentrations in the urine samples were determined based on neat solvent calibration functions established between 0.3 and 100 ng/mL, considering matrix effects (which ranged between 84% and 115% for all analytes) and the dilution factor.

The D–S approach was also applied for sample preparation of pig urine samples was to analyse ZEN and its metabolites (α-ZEL, β-ZEL, ZEN-14-Glc, ZEN-16-Glc, ZEN-14-S, α-ZEL-GlcAc, β-ZEL-GlcAc, ZEN-14-GlcAc) [56]. Before analysis, all urine samples were diluted to the same creatinine concentration (0.2 mM). The D–S approach resulted in similar LODs/LOQs for ZEN (0.15/0.49), α-ZEL (0.11/0.38), β-ZEL (0.16/0.54 ng/mL) compared to SPE columns and much higher compared to a combination of IAC and SPE columns. The apparent recovery ranged from 84% for β-ZEL to 113% for α-ZEL-GlcAc.

Although D–S is time- and cost-effective for sample preparation, where the urine sample is diluted and injected directly into the LC-MS/MS system, the latest state-of-the-art triple-quadrupole mass analyser is needed to achieve the very low LODs.

The most popular sample preparation for analysing multiple mycotoxins (not only regulated in feed) in pig urine samples is based on liquid-liquid extraction. LLE with acetonitrile as extraction solvent was for the first time applied for the determination of T-2, HT-2 and T-2 triol [51]. The LODs for T-2, HT-2, and T-2 triol were 0.3, 0.6, and 2 ng/mL, and the LOQs were 1, 2, and 5 ng/mL, respectively. The extraction recovery rates ranged from 88.3% for HT-2 to 100.0% for T-2 triol.

Two extraction protocols for pig urine, i.e., one in acidic medium (pH 2) and another in neutral medium (pH 7) with ethyl acetate as extraction solvent, were developed to extract 24 mycotoxins including ZEN, DON, T-2, AFB_1_, their metabolites and additionally: OTA, FB_1_, Alternaria toxins (TeA, AOH, AME), ENNs and BEA [64]. A high-resolution mass spectrometer (HRMS) was used to determine the phase I and II metabolite, for which no commercial analytical standards were available. Therefore, enzymatic hydrolysis was not performed in this study. The LOQ was 1 ng/mL for most of the compounds, with the following exceptions: DOM-1 (4 ng/mL) and T-2 toxin-di-glucoside (T-2-Glc) (2 ng/mL). Most analytes gave acceptable results (range 60–140%). However, for some mycotoxins, matrix effects were more pronounced, and recovery was relatively low. Therefore, for all mycotoxins, an adequate internal standard and matrix-matched calibration curves were used, resulting in validation results for accuracy and precision matching the acceptance criteria.

Two other published multi-biomarker methods used LLE with some modifications [77,78]. In the first study, a novel clean-up method based on an impurity adsorption mechanism has been developed to purify 25 mycotoxins and their metabolites (in particular: DON, ZEN, AFB1, T-2, their metabolites and other mycotoxins: STC, DAS, LYS, MET, RC, NEO, WOR, VER in animal urine. 0.1% formic acid- acetonitrile solution was added after the addition of 0.8 g of sodium chloride. After vortexing and centrifuging upper supernatant was additionally mixed with 500 mg of anhydrous magnesium sulfate, 50 mg of C18, 50 mg of primary, secondary amine (PSA), and 50 mg of alumina A and then the supernatant was evaporated to dryness and resolved in the mobile phase [78]. The LOQ values ranged from 0.05 ng/mL for AFs, STC, LYS, T-2, MET, RC, DAS to 0.5 ng/mL for α,β-ZEL/ZAL and ZAL. The recoveries of target analytes in pig-urine samples varied from 80.8% for α-ZEL to 114.3% for β-ZAL. Significant signal suppression was noticed for most compounds such as AFM_1_, DAS, DON, 15-AcDON and 3-AcDON etc. Therefore, matrix-matched calibration standard curves were selected to quantify target compounds in urine. Although, internal standard correction was not applied in this study.

In the second study, a method based on salting-out assisted liquid-liquid extraction (SALLE) procedure was developed and validated for simultaneous analysis of AFB_1_, DON, FB_1_, OTA, ZEN and T-2 and their metabolites (in total 12 analytes) in pig urine [77]. The salt MgSO_4_ (2 M) and ethyl acetate/FA (99/1, *v*/*v*) were applied as the first extraction solvent, and acetonitrile/FA (99/1, *v*/*v*) was added to the remaining aqueous phase. After extraction, the acetonitrile phase was combined with the ethyl acetate phase, evaporated and reconstituted in the mobile phase. The LOD/LOQ ranged from 0.02/0.07 ng/mL for OTA to 1/3.3 ng/mL for DON. The extraction recoveries were in a range of 70% for DON to 108% for T-2. Significant signal suppression was noticed for all analytes. Therefore, matrix-matched calibration was used for quantification. Although, an internal standard correction was not applied in this study. Additionally, SALLE and D–S approaches were compared. For the D–S method, much more severe signal suppression was observed for the D–S method, and high concentration of the analytes is required for a significant signal to be seen. In some extreme cases (AFM1), the LODs were 8 and 20 times higher with D–S than with SALLE. SALLE method was also validated for human urine and resulted in two timed lower LODs/LOQs values.

The described LLE methods enabled a determination of a wide range of mycotoxins (up to 25) in pig urine samples. The methods, which applied SPE columns or were based on the D–S approach, allowed determination of only DON, ZEN, and metabolites. IAC columns with a combination of SPE columns additionally enabled determination of AFM_1_, OTA, FB_1_. As an extraction solvent in LLE, ACN and EtOAc or their acidified solution with 1% FA and salts additions were used to extract multiple mycotoxins from urine samples. Using impurity adsorption purification, lower LOQs compared to other LLE methods were achieved. However, significant signal suppression was observed for the majority of mycotoxins. In general, LLE allowed to develop sensitive (LOQ ≤ 1 ng/mL for most analytes) and multi mycotoxin (more than 20 analytes) methods; although, significant steps such as enzymatic hydrolysis and creatinine adjustment were missing in the advanced LLE methods.

Recently, a novel method for determining 35 mycotoxins (nivalenol, citrinin, dihydrocitrinone, fusarenon-X, altertoxin I, tentoxin and hydrolysed fumonisin B1 were quantified in pig urine samples for the first time) in pig urine samples was developed [88]. Sample preparation includes creatinine adjustment (with the developed LC-UV method) with enzymatic hydrolysis of pig urine samples followed by LLE. The LLE protocol and enzymatic hydrolysis for indirect mycotoxin glucuronides determination were optimised in this study. The method was validated concerning the guidelines specified by the EMEA (European Medicines Agency). The extraction recoveries were higher than 60% for 77% of the analytes studied. The intra- and inter-day relative standard deviations were lower than 20% for most compounds at four different concentration levels. The LOQs ranged from 0.1 ng/mL for zearalenone and sterigmatocystin to 8 ng/mL for nivalenol. Additionally, the matrix effect was evaluated for the first time in this study for six different urine samples. The coefficient of variation was lower than 15% for most analytes studied.

### 4.2. Faeces

To extract multiple mycotoxins from pig faeces, two or three steps LLE with different solvents was needed. Application of MeOH/ H_2_O (50/50, *v*/*v*) for determination of DON, D3G and DOM-1 resulted in good extraction recovery 85–95% and significant signal suppression. LOD/LOQ values were 3.4/11.2 ng/g for DON, 2.6/8.7 ng/g for D3G and 3.2/10.8 ng/g for DOM-1. The mixture of ACN/water (50/50, *v*/*v*) was applied as an extraction solvent to determine ZEN and its metabolites (α-ZEL, β-ZEL, ZEN-14-Glc, ZEN-16-Glc and ZEN-14-S) in pig faeces. Extraction recovery ranged from 103% to 139%. Significant signal suppression was noticed only for α-ZEL (47%). LOD/LOQ ranged from 0.06/0.19 ng/g for ZEN-14-Glc to 0.73/2.4 ng/g for ZEN-16-Glc. T-2, HT-2 and T-2 triol were extracted from pig faeces with EtOAc and then additionally, the supernatants were applied to a Varian Bond-Elut Mycotoxin SPE cartridge. Extraction recoveries ranged from 74.3% to 102.4%. The LOD/LOQ values were 0.3/1 ng/g for T-2, 0.6/2 ng/g for HT-2 and 2/5 ng/g for T-2 triol.

In another multi-marker method (24 mycotoxins), the mixture MeOH/EtOAc/FA (75/24/1; *v*/*v*) was applied to extract OTA, TeA, AME, and AOH. After extraction with acetone and additional clean-up with a solid-phase extraction with a HybridSPE-phospholipid column, other mycotoxins (ZEN, DON, T-2, AFB_1_, their metabolites, ENNs and BEA) were determined in pig faeces. The extraction recoveries ranged from 41.2% to 266.1%. Significant signal suppression was noticed for a majority of analytes except ENNA_1_—effective signal suppression. The LOQ values ranged from 1 ng/g to 5 ng/g.

### 4.3. Serum

Recently, LLE with ACN as an extraction solvent has been the most popular sample preparation protocol for pig plasma samples to determine multiple mycotoxins and metabolites. In particular, it was applied for determination of DON, ZEN, T-2, OTA, FB_1_, AFB_1_ and their metabolites and resulted in very low LODs/LOQs: for DON and its metabolites (0.01/0.1–0.2/0.1 ng/mL) [89], ZEN and its metabolites (0.003/0.2–0.04/5 ng/mL) [90], T-2 and its metabolites (0.3/1–2/5 ng/mL) [51], ENNs and BEA (0.001/0.1–0.01/0.2 ng/mL) [91] as well as multi-mycotoxin method for simultaneous determination of DON, ZEN, T-2, their metabolites, OTA, FB_1_ and AFB_1_ (0.01/1–0.4/10 ng/mL). Enzymatic hydrolysis was applied in only one method for ZEN and its metabolite determination, [90]. Although, as shown in Table 3, mycotoxins are excreted in pig plasma, mainly in free form, the degree of conjugation of DON in serum found in the previous study was approximately 33% (19–45%) [54]. Good extraction recoveries (74.3–109.8%) were found in every abovementioned method except for 15-AcDON (64.8%) [89]. Neither significant signal suppression nor enhancement was noticed in these methods. In another multi-mycotoxin method for determination of 24 mycotoxins (DON, ZEN, AFB_1_, T-2, their metabolites, TeA, AOH, AME, ENNs and BEA) with the LLE procedure, acidified ACN (0.1% FA) was applied as the extraction solvent. In those methods, significant signal suppression (SSE < 80%) was noticed for 10 of the analytes [64].

SPE columns (Oasis HLB) were applied once in pig plasma analysis of DON, ZEN and their metabolites after β-glucuronidase pretreatment [61]. The determined LOD and LOQ for the quantifier ions of all analytes ranged 0.03–0.71 ng/mL and 0.08–2.37 ng/mL, respectively. Good recoveries (85–117%) were obtained for all analytes.

## 5. Conclusions

Pigs are the most sensitive species to mycotoxins, and most of the established TDI for humans was based on adverse effects in pigs. Although LOAELs are established for DON, ZEN, OTA, FB_1_ and T-2, there is a lack of toxicological data to establish NOAELs. NOAELs exist only for DON and ZEN. LOAELs should also be verified in more in vivo studies because most of those studies are ancient and unreliable, as shown in the FB_1_ example. Guidance values exist only for pig feed. The lack of guidelines for risk levels in physiological samples makes it impossible to interpret the results of biomarker studies without feed analysis.

The most researched *Fusarium* mycotoxins in pigs in vivo trials are DON and ZEN. For these analytes, suitable biomarkers in all biological matrices are selected and detailed described in contrast to other (regulated in feed) mycotoxins. Additionally, feed is frequently contaminated with “mycotoxin mixture”. Therefore, multi-biomarker studies should be performed to reflect the actual exposure of the pigs to mycotoxins.

Analysis of biomarkers should be performed only with reliable analytical methods. Multi-mycotoxin direct approaches (D–S) designed to monitor pig exposure to DON, ZEN and its metabolites in urine are generally less sensitive for DON, ZEN and its metabolites than indirect analytical methods using enzymatic hydrolysis and SPE, IAC or LLE. In general, LLE allowed to develop of sensitive (LOQ ≤ 1 ng/mL for a majority of analytes) and multi mycotoxin (more than 20 analytes) methods in all pig biological matrices.

Although biomarkers are essential and valuable tools in scientific studies, more knowledge is needed before biomarkers can be used in practice on farms. The choice of suitable biomarker, matrix, and sampling time plays the most important role in adequate exposure assessment. Due to the range of metabolites resulting from a single mycotoxin and the differences in toxicity, biomarkers as a diagnostic tool are only possible within scientific trials.

## Figures and Tables

**Table 1:** NOAEL (no-observed-adverse-effect level) and LOAEL (lowest-observed-adverse-effect level) levels of selected mycotoxins recommended by EFSA.

Mycotoxin	Pig Type	Adverse Effect	LOAEL	NOAEL	Ref.
DON	-	vomiting	2.8 mg/kg feed	0.7–12 mg/kg feed	[17]
		reduced feed intake and weight gain	0.35 mg/kg feed	0.7 mg/kg feed	
FB_1_	-	lung lesions	0.4 mg/kg b.w./day	-	[20]
		increased serum Sa:So (sphinganine:sphingosine) ratios	5 mg /kg feed (0.2 mg/kg b.w./day	-	
OTA	-	progressive nephropathy	1 mg/kg feed (40 µg/kg b.w.)	-	[19]
		effects on renal (diagnostic) enzyme levels and kidney function	0.2 mg/kg feed (8 µg/kg b.w.)	-	
T-2	-	immunological or haematological effects	0.2 mg/kg feed (8 µg/kg b.w.)	-	[21]
ZEN	female prepubertal piglets	oestrogenic effects such as increased uterus weight and reddened and swollen vulva	17.6 µg/kg b.w./day	10.4 µg/kg b.w./day	[18]
	sexually mature female pigs	prolonged cycling	200 µg/kg b.w./day	40 µg/kg b.w./day	

DON—deoxynivalenol, FB_1_—fumonisin B_1_, OTA—ochratoxin A, T-2—T-2 toxin, ZEN—zearalenone.

**Table 2:** Summary of recent (after year 2000) ZEN dose-respond studies in pigs.

ZEN Feeding Group	Dose [µg/kg Feed]	Feed Type	Matrix 1	Biomarker	Biomarker Concentration (ng/mL)	Matrix 2	Biomarker	Biomarker Concentration (ng/mL)	Experiment Time	Number and Type of Pig	Ref.
1	4	naturally contaminated wheat	urine	ZEN α-ZEL	10.4 16.4	serum	-	-	35 days	12–180 days old gilts	[54]
2	88				16.7 18.3						
3	235				15.4 22.1						
4	358				43.1 57.9						
1	10	naturally contaminated maize	urine	ZEN α-ZEL	2.7 ** 1.5	-	-	-	37 days	100 female piglets	[48]
2	60				34.8 10.2						
3	150				31.5 9.9						
4	220				64.1 20.3						
1	350	naturally contaminated maize	urine	ZEN ZEN-GlcAc α-ZEL α-ZEL-GlcAc	-	-	-	-	28 days	24 castrated male pigs	[55]
2	720										
3	1680										
4	2360										
1	10	contaminated maise	urine	ZEN α-ZEL β-ZEL ZAN α-ZAL	3.4 * (0.5–11.4) 0.6 (<LOQ–35.2) <LOQ <LOQ <LOQ	serum	ZEN α-ZEL	0 0	29 days	125 female weaned piglets	[47]
2	50				25.9 (1.0–82.0) 9.7 (0–22.7) <LOQ <LOQ <LOQ			0–0.1 0			
3	80				51.7 (1.1–122) 18.6 (0–63.2) 1.4 (<LOQ–18.2) 0.4 (<LOQ–1.4) <LOQ			0–0.2 0			
4	170				79 (9.5–237) 35.1 (2.3–89.2) 10.2 (<LOQ–42.1) 0.8 (<LOQ–2.3) <LOQ			0–0.3 0–0.8			
5	290				136 (7.7–327) 57.2 (2.4–122) 15.1 (0–42.9) 1.7 (0–3.4) 0.5 (0–0.7)			0–0.5 0–1.6			
1	6.04	feed boluses fortified with pure cultures	urine	ZEN α-ZEL β-ZEL	11.63 ± 7.52 3.60 ± 3.70 nd	-	-	-	3 days	16 weaned piglets	[49]
2	17.23				23.06 ± 11.42 6.76 ± 4.43 nd						
3	21.13				15.76 ± 9.62 5.24 ± 3.29 nd						
4	50.26				17.08 ± 4.15 6.58 ± 2.36 0.39 ± 0.36						

ZEN—zearalenone, ZEN-GlcAc—zearalenone glucuronide, α-ZEL—α-zearalenol, β-ZEL—β-zearalenol, ZAN—zearalanone, α-ZAL—α-zearalanol, β-ZAL—β-zearalanol, α-ZEL-GlcAc—α-zearalenol glucuronide. * median concentration, ()–range, ** mean concentration

**Table 3:** Summary of recent (after year 2000) DON dose-respond studies in pigs.

Feeding Group	Dose (µg/kg Feed)	Feed Type	Matrix 1	Biomarker	Biomarker Level (ng/mL)	Matrix 2	Biomarker	Biomarker Level (ng/mL)	Experiment Time	Number and Type of Pig	Ref.
1	210	contaminated wheat	urine	DON DOM-1	299 29	serum	DONDOM-1	1 0.1	35 days	12–180 days old gilts	[54]
2	3070				690 150			4.1 1.3			
3	6100				804 177			14.3 2.8			
4	9570				1572 289			21.6 4.1			
1	200	contaminated maize	urine	DON DOM-1	56 <LOD	serum	DONDOM-1	<LOQ	37 days	100 female piglets	[48]
2	800				162 <LOD			1 (<LOQ-4)			
3	1000				360 14			5 (<LOQ-12)			
4	1900				246 14			6 (<LOQ-13)			
5	3900				380 19			11 (6–19)			
1	1110	contaminated maize	urine		-	-	-	-	28 days	24 castrated male pigs	[55]
2	2320										
3	3700										
4	5000										
1	30	contaminated maize	urine	DONDOM-1	68.3 (18.6–231) 20.4 (5.3–171)	serum	DONDOM-1	0.5 (0–1.4) 0	29 days	125 female weaned piglets	[47]
2	590				524 (50.8–1070) 43.4 (1.8–140)			4.9 (2.7–7.9) 0			
3	1270				1065 (96.2–2120) 70.3 (1.6–336)			8.6 (4.6–15.9) 0			
4	2010				1850 (288–4050) 118 (1.2–513)			15.2 (8.1–24.9) 2.9 (0–4.0)			
5	4520				2680 (244–4990) 262 (0.7–979)			24.4 (9.9–42.8) 4.5 (0–7.4)			
1	63.58	fortified feed boluses	urine	DONDOM-1	80.55 ± 28.96 1.19 ± 1.38	-	-	-	3 days	16 weaned piglets	[49]
2	181.51				125.06 ± 41.64 3.36 ±1.35						
3	214.36				305.94 ± 143.17 3.44 ± 4.14						
4	509.53				218.18 ± 33.35 16.33 ± 5.95						

DON—deoxynivalenol, DOM-1—deepoxy-deoxynivalenol.

**Table 4:** Summary of recent (after year 2000) AFB_1_, OTA, FB_1_ and T-2 dose-respond studies in pigs.

Mycotoxin	Feeding Group	Dose	Feed Type	Matrix 1	Biomarker	Biomarker Level (ng/mL)	Matrix 2	Biomarker	Biomarker Level (ng/mL)	Experiment Time	Number and Type of Pig	Ref.
AFB_1_	1	0.16 µg/kg b.w.	feed boluses fortified with pure cultures	urine	AFM_1_	0.14	-			3 days	16 weaned piglets	[49]
	2	0.45 µg/kg b.w.				0.36						
	3	0.54 µg/kg b.w.				0.50						
	4	1.28 µg/kg b.w.				0.88						
	1	127 µg/kg feed	pure crystal AFB_1_ dissolved in methanol (20 mL) and sprayed on 2 kg of feed	urine	AFM_1_ AFB_1_ AFB_2_	12.6 9.9 0.64	-			18 days	4 castrated male pigs	[50]
	2	227 µg/kg feed				17.2 1.9 0.76						
	3	327 µg/kg feed				22.6 3.7 0.51						
OTA	1	0.16 µg/kg b.w.	feed boluses fortified with mycotoxins	urine	OTA	0.12	-			3 days	16 weaned piglets	[49]
	2	0.46 µg/kg b.w.				0.65						
	3	0.56 µg/kg b.w.				0.52						
	4	1.32 µg/kg b.w.				0.36						
OTA	1	50 µg/kg feed	basal diet mixed with pure OTA standard solutions	plasma	OTA	22.2 ± 2.6	-			15 days	12 pigs	[65]
	2	500 µg/kg feed				217.4 ± 25.1						
	1	25 µg/kg feed	crystalline OTA	urine	OTA	3.1–4.35	-			119 days	24 pigs	[66]
	1	800 µg/kg feed	naturally contaminated feed	serum	OTA	852–1582	-			180 days	6 pigs	[67]
	1	120 µg/kg feed	naturally contaminated feed	plasma	OTA	82.8				28 days	48 piglets	[68]
	1	250 µg/kg feed	OTA-fortified feed	urine	OTA	16.1	serum	OTA	4.8	28 days	10 pigs	[69]
	1	300 µg/kg feed	pure OTA standard mixed with lactose and formulated as gelatine capsules	plasma	OTA	6.4				30 days	10 pigs	[70]
	1	114 µg/kg feed	naturally contaminated feed	urine	OTA OTα	18.8 ± 6.4 9.5 ± 2.6	serum	OTA OTα	141 ± 47.9 < LLOQ	28 days	24 pigs	[71]
	2	226 µg/kg feed				36.5 ± 11.6 16.2 ± 5.5			278 ± 106 0.69 ± 0.10			
FB_1_	1	3.71 µg/kg b.w.	feed boluses fortified with pure cultures	urine	FB_1_	1.55	-			3 days	16 weaned piglets	[49]
	2	10.6 µg/kg b.w.				3.36						
	3	64.2 µg/kg b.w.				77.37						
	4	150 µg/kg b.w.				117.78						
	1	5 mg/kg b.w.	aqueous stock solutions	urine	FB_1_	-	faeces	FB_1_	-	96 h	male weaned piglets	[72]
	1	3.1 µg/g	culture material added to feed	urine	FB_1_	16.09 ± 21.94	plasma	FB_1_	0.16 ± 0.04	28 days	24 piglets	[73]
	2	6.1 µg/g				24.08 ± 25.96			0.26 ± 0.06			
	3	9.0 µg/g				18.88 ± 4.41			0.42 ± 0.10			
FB_1_ FB_2_ FB_3_	1	50 mg/animal	*F.verticillioides* fungal culture mixed into feed	urine (13–17 day)	FB_1_ FB_2_	4.5 ± 3.9 mg 0.5 ± 0.5 mg	faeces	FB_1_FB_2_	28.2 ± 27.3 mg 2 ± 1.1 mg	22 days	6 cross-bred pigs (plasma) /15 weaned barrows (urine)	[52]
	2	20 mg/animal										
	3	5 mg/animal										
T-2	1	500	aqueous stock solutions	urine	T-2 HT-2 T-2 triol	30.9 ± 2.1 614.4 ± 177 306 ± 70	faeces	HT-2	104.6 ± 14.2 (36 h)	1 day	7 cross-bred pigs	[51]

AFB_1_—aflatoxin B_1_, AFB_2_—aflatoxin B_2_, AFM_1_—aflatoxin M_1,_ OTA—ochratoxin A, OTα—ochratoxin alpha. FB_1_—fumonisin B_1_, FB_2_—fumonisin B_2_, T-2—T-2 toxin, HT-2—HT-2 toxin.

**Table 5:** The essential biomarkers (with adequate sampling time in brackets (when studied)) found in pig biological matrices in vivo studies with pigs (in bold are biomarkers found at the highest concentration).

Mycotoxin/Matrix/Time	Urine	Faeces	Plasma
DON	DON (4–8 h) DOM-1 DON-GlcAc	DOM-1 (8–24 h) DON	DON-3-GlcAc (3–4 h) DON * (3–4 h) DOM-1
ZEN	ZEN-GlcAc (12–24 h) ZEN * (12–24 h) α-ZEL β-ZEL ZAN α-ZAL β-ZAL	ZEN (12–24 h) α-ZEL (12–24 h) ZAN	ZEN-GlcAc (0.25–3 h) ZEN * (0.25–3 h) α-ZEL (0.25–4 h)
OTA	OTA OTα	n.d.	OTA (72–120 h) OTα
FB_1_/FB_2_	FB_1_ (8–24 h) FB_2_	FB_1_ (8–24 h) FB_2_	FB_1_ (2 h)
AFB_1_	AFM_1_ AFB_1_ AFB_2_	n.d.	n.d.
T-2	HT-2 (12–24 h) T-2 triol (12–24 h) T-2	HT-2	T-2 (3 h)

* analysis after enzymatic hydrolysis. DON—deoxynivalenol, DOM-1—deepoxy-deoxynivalenol, DON-GlcAc—deoxynivalenol glucuronide, ZEN—zearalenone, ZEN-GlcAc—zearalenone glucuronide, α-ZEL—α-zearalenol, β-ZEL—β-zearalenol, ZAN—zearalanone, α-ZAL—α-zearalanol, β-ZAL—β-zearalanol, OTA—ochratoxin A, OTα—ochratoxin alpha, FB_1_—fumonisin B_1,_ FB_2—_fumonisin B_2,_ AFB_1_—aflatoxin B_1_, AFB_2_—aflatoxin B_2_, AFM_1_—aflatoxin M_1_, T-2—T-2 toxin, HT-2—HT-2 toxin, T-2 triol—T-2 toxin triol.

## Data Availability

Not applicable.

## Supplementary Materials

The following are available online at https://www.mdpi.com/article/10.3390/toxins13080586/s1, Table S1: Summary of recent LC-MS/MS methods for determination of mycotoxin biomarkers in pig urine, faeces and serum.
