# Supplementary material for: Mycotoxin Biomarkers in Pigs—Current State of Knowledge and Analytics
Source: Toxins (Basel). 2021 Aug 23;13(8):586. doi: 10.3390/toxins13080586 (PMC8402396; doi:10.3390/toxins13080586)
Supplement: Supplementary file 1 [file toxins-13-00586-s001.zip › toxins-1327037-SI .pdf]

Mycotoxin Biomarkers in Pigs—Current State of Knowledge and Analytics

Agnieszka Tkaczyk * and Piotr Jedziniak

Table S1. Summary of recent LC-MS/MS methods for determination of mycotoxin biomarkers in pig urine, faeces and serum

Matrix	Sample preparation		Creatinine adjustment	Enzymatic hydrolysis (enzyme)	Analytes	LOD [ng/mL]	LOQ [ng/mL]	Recovery [%]	Matrix effect (SSE) [%]	Analytical column	Mobile phase	LC-MS	Reference
urine	SPE columns	RP-18 SPE (Phenomenex)	-	glucuronidase/ arylsulfatase from Helix pomatia	ZEN<br>α-ZEL<br>β-ZEL<br>α-ZAL<br>β-ZAL	0.1<br>0.2<br>0.2<br>0.5<br>0.5	0.5<br>0.5<br>0.5<br>1<br>1	94-105	-	RP- 18 column	10% ACN, 45% MeOH , 45% H_2O (v/v/v)	PE Sciex API 365 (APCI)	[1]
urine	SPE columns	Oasis™ HLB columns (Waters)	-	type H-2 from Helix pomatia	ZEN<br>α-ZEL<br>β-ZEL<br>ZAN<br>α-ZAL<br>β-ZAL<br>DON<br>DOM-1	0.03<br>0.07<br>0.16<br>0.09<br>0.11<br>0.13<br>0.11<br>0.04	0.10<br>0.24<br>0.52<br>0.30<br>0.35<br>0.44<br>0.38<br>0.15	76-118	-	-	-	1200 series HPLC system (Agilent Technologies) coupled to a 4000 QTrap (Applied Biosystems)	[2]
urine	Myco6in1 immunoaffinity (Vicam) column (IAC) (Vicam) and an OASIS HLB solid phase extraction (SPE) column (Waters) connected in tandem		-	β-glucuronidase/ sulfatase type H-2 from Helix pomatia	DON<br>DOM-1<br>AFM_1<br>FB_1<br>ZEN<br>α-ZEL<br>β-ZEL<br>OTA	0.18<br>0.36<br>0.01<br>0.02<br>0.02<br>0.04<br>0.04<br>0.006	0.6<br>1.21<br>0.03<br>0.06<br>0.07<br>0.13<br>0.15<br>0.02	64-100	12<br>18<br>33<br>152<br>44<br>14<br>29<br>42	-<br>-	-<br>MeOH:H2O, 20:80, v/v)	QTrap MS/MS system (Applied Biosystems, Foster City, CA, USA), equipped with an ESI interface, an 1100 series micro-LC system	[3]<br>[4]
urine	D&S		-	-	DON<br>DON-3-Glc<br>DON-3-GlcA<br>DOM-1	0.9<br>1.3<br>9.0<br>1.4	8<br>2.1<br>37.3<br>3.7	94-103 (R_E)<br>56-114 (R_A)	56<br>62<br>72<br>122	Zorbax RRHD Eclipse XDB-C18 column Agilent Technologies ,Waldbronn, Germany)	Water and MeOH/water (97/3, v/v), both containing 0.01% acetic acid and 5 mM ammonium acetate	1290 Infinity series UHPLC system (Agilent Technologies, Waldbronn, Germany) coupled to a 4000 QTrap mass spectrometer	[5]

Matrix	Sample preparation	Creatinine adjustment	Enzymatic hydrolysis (enzyme)	Analytes	LOD [ng/mL]	LOQ [ng/mL]	Recovery [%]	Matrix effect (SSE) [%]	Analytical column	Mobile phase	LC-MS	Reference
urine	D&S	urine diluted to the same creatinine concentration (0.2 mM)	-	DON<br>DON-GlcAc<br>DON-3-S<br>DOM-1<br>ZEN<br>ZEN-14-GlcAc<br>α-ZEL<br>α-ZEL-14-GlcAc	-	-	84- 115	-	Kinetex Biphenyl column (150×3 mm, 2.6-μm particle size; Phenomenex, Aschaffenburg, Germany)	water/acetic acid and methanol/ acetic acid (each 99.9/0.1, v/v)	Agilent 1290 series UHPLC system coupled to a Sciex 6500 QTrap mass spectrometer	[6]
				α-ZEL<br>β-ZEL<br>ZEN<br>ZEN-14-Glc<br>ZEN-16-Glc<br>ZEN-14-S<br>α-ZEL-GlcAc<br>β-ZEL-GlcAc<br>ZEN-14-GlcAc	0.11<br>0.16<br>0.15<br>0.07<br>0.18<br>0.02<br>0.73<br>0.42<br>0.30	0.38<br>0.54<br>0.49<br>0.23<br>0.60<br>0.08<br>2.4<br>1.4<br>1.0	84-113 (R_A)	-	Kinetex Biphenyl column (150 mm × 3 mm, 2.6 μm, Phenomenex, Aschaffenburg, Germany)	water/acetic acid (99.9/0.1, v/v), and ACN/acetic acid (99.9/0.1, v/v)	Agilent 1290 series UHPLC system coupled to a Sciex 6500 QTrap mass spectrometer	[7]
	LLE (ACN)	-<br>-	-	T-2<br>HT-2<br>T-2 triol	0.3<br>0.6<br>2	1<br>2<br>5	74.3- 102.4	79.3-93.8	Zorbax XDB-C18 column (150 mm × 2.1 mm, 3.5 μm; Agilent, USA)	5.0 mM ammonium acetate in water (A) and acetonitrile (B)	1290 Infinity series UHPLC system (Agilent Technologies, Waldbronn, Germany) coupled to a 4000 QTrap mass spectrometer	[8]
**Matrix**	**Sample preparation**	**Creatinine adjustment**	**Enzymatic hydrolysis (enzyme)**	**Analytes**	**LOD [ng/mL]**	**LOQ [ng/mL]**	**Recovery [%]**	**Matrix effect (SSE) [%]**	**Analytical column**	**Mobile phase**	**LC-MS**	**Reference**
urine	LLE<br>EtOAc	-	-	ZEN<br>α-ZEL<br>α-ZAL	-	1 for all analytes except:	56.2 (T-2)-212.8 (AOH) (R_E)	90.6<br>84.8<br>85.8	Waters Acquity HSS T3	10 mM ammonium formate,	Agilent 1290 series UHPLC system coupled	[9]<br><br>[10]

	β-ZAL	DOM-1	85.1	column (100	0.3% formic	to a Sciex 6500
	β-ZEL	(4 ng/mL)	85.5	mm × 2.1	acid in	QTrap mass
	ZAN	and	90.6	mm i.d., dp:	water and	spectrometer
	TeA	T2G	52.4	1.8 μm)	10 mM	
	AOH	(2 ng/mL)	11.1		ammonium	
	AME		73.6		formate,	
	DON		79.5		0.3% formic	
	DOM-1		5.2		acid in	
	3/15-		72.6		methanol	
	AcDON		90.5			
	T-2		100.4			
	HT-2		107.6			
	T2-G		72.4			
	AFB_1		79.3			
	AFM_1		106.3			
	OTA		81.3			
	ENNA		80.2			
	$ENNA_1$		83.7			
	ENNB		82.8			
	$ENNB_1$		89.5			
	BEA					

Matrix	Sample preparation	Creatinine adjustment	Enzymatic hydrolysis (enzyme)	Analytes	LOD [ng/mL]	LOQ [ng/mL]	Recovery [%]	Matrix effect (SSE) [%]	Analytical column	Mobile phase	LC-MS	Reference
urine	LLE<br>1. step :<br>0.1% formic acid-ACN + 0.8 g of sodium chloride.<br>2. step : upper supernatant + 500 mg of anhydrous magnesium sulfate, 50 mg of C18, 50 mg of PSA, and 50 mg of aluminia A	-	-	AFB_1<br>AFB_2<br>AFG_1<br>AFG_2<br>AFM_1<br>AFM_2<br>STC<br>T-2<br>LYS<br>MET<br>RC<br>DAS<br>DON<br>3-AcDON<br>15-AcDON<br>NEO<br>WOR<br>VER<br>HT-2<br>ZEN<br>α-ZEL<br>β-ZEL<br>ZAN<br>α-ZAL<br>β-ZAL	-	0.05<br>0.05<br>0.05<br>0.05<br>0.05<br>0.05<br>0.05<br>0.05<br>0.05<br>0.05<br>0.05<br>0.05<br>0.25<br>0.5<br>0.5<br>0.25<br>0.25<br>0.25<br>0.25<br>0.25<br>0.5<br>0.5<br>0.5<br>0.5<br>0.5	80.8 - 114.3	signal suppression for the majority of compounds	BEH RP18 chromatographic column (Acquity UPLC, 100 mm × 2.1 mm; 1.7 µm; Waters, USA)	0.1% formic acid aqueous solution and 0.1% formic acid-methanol	Waters Acquity ultra-performance liquid chromatography (UPLC) system coupled to a Micromass Quatro Micro triple quadrupole mass spectrometer (Waters, Milford, MA, USA)	[11]
	LLE<br>1. step:<br>EtOAc/FA (99/1, v/v) + $MgSO_4$ (2 M)<br>2. step: remaining aqueous phase+ ACN/FA (99/1, v/v).	-	-	DON<br>AFB_1<br>AFM_1<br>T-2<br>HT-2<br>NEO<br>FB_1<br>OTA<br>OTα<br>ZEN<br>α-ZEL<br>β-ZEL	1<br>0.1<br>0.1<br>0.04<br>0.3<br>1<br>0.05<br>0.02<br>0.25<br>0.4<br>0.3<br>0.3	3.3<br>0.33<br>0.33<br>0.13<br>3.3<br>0.17<br>0.07<br>0.8<br>1<br>1.3<br>1<br>1	70-100	4<br>5<br>5<br>32<br>17<br>4<br>57<br>46<br>4<br>20<br>23<br>16	Symmetry C18, 5 µm, 2.1×150 mm (Waters, Milford, MA, USA)	water, 0.3% formic acid, 5mM ammonium format and methanol, 0.3% formic acid, 5mM ammonium formate	Waters Acquity ultra-performance liquid chromatography (UPLC) system coupled to a Micromass Quatro Micro triple quadrupole mass spectrometer (Waters, Milford, MA, USA)	[12]

**Matrix**	**Sample preparation**	**Creatinine adjustment**	**Enzymatic hydrolysis (enzyme)**	**Analytes**	**LOD [ng/mL]**	**LOQ [ng/mL]**	**Recovery [%]**	**Matrix effect (SSE) [%]**	**Analytical column**	**Mobile phase**	**LC-MS**	**Reference**
faeces	extraction **MeOH/ water (50/50, v/v, 3/2/2 mL** x3	-	-	DON<br>DON-3-Glc<br>DOM-1	3.4<br>2.6<br>3.2	11.2<br>8.7<br>10.8	85-95 (RE)<br>52-59 (RA)	63<br>62<br>59	Zorbax RRHD Eclipse XDB-C18 column Agilent Technologies ,Waldbronn, Germany)	Water and MeOH/water (97/3, v/v), both containing 0.01% acetic acid and 5 mM ammonium acetate	1290 Infinity series UHPLC system (Agilent Technologies, Waldbronn, Germany) coupled to a 4000 QTrap mass spectrometer	[5]
	extraction **ACN/water (50/50, v/v) x3**	-	-	α-ZEL<br>β-ZEL<br>ZEN<br>ZEN-14-Glc<br>ZEN-16-Glc<br>ZEN-14-S	.0.08<br>0.30<br>0.10<br>0.06<br>0.73<br>0.21	0.28<br>1.0<br>0.33<br>0.19<br>2.4<br>0.7	103-139 (RE)<br>49 - 124 (RA)	47<br>75<br>77<br>66<br>75<br>104	Kinetex Biphenyl column (150 mm × 3 mm, 2.6 µm, Phenomenex, Aschaffenburg, Germany)	water/acetic acid (99.9/0.1, v/v), and ACN/acetic acid (99.9/0.1, v/v)	1290 Infinity series UHPLC system (Agilent Technologies,Waldbronn, Germany) coupled to a 6500 QTrap mass spectrometer (Sciex, Foster City, CA, USA)	[7]
	extraction (EtAc) x2 + SPE columns			T-2<br>HT-2<br>T-2 triol	0.3<br>0.6<br>2	1<br>2<br>5	74.3-102.4 (RE)	79.3-93.8	Zorbax XDB-C18 column (150 mm × 2.1 mm, 3.5 µm; Agilent, USA)	5.0 mM ammonium acetate in water and acetonitrile	1290 Infinity series UHPLC system (Agilent Technologies, Waldbronn, Germany) coupled to a 4000 QTrap mass spectrometer	[8]

Matrix	Sample preparation	Creatinine adjustment	Enzymatic hydrolysis (enzyme)	Analytes	LOD [ng/mL]	LOQ [ng/mL]	Recovery [%]	Matrix effect (SSE) [%]	Analytical column	Mobile phase	LC-MS	Reference
faeces	**1.LLE MeOH/ethyl acetate/formic acid (75/24/1; v/v)** for OTA, TeA, AME and AOH.<br><br>2. the other mycotoxins a **LLE with acetone and a solid phase extraction with a HybridSPE-phospholipid column**	-	-	ZEN<br>α-ZEL<br>α-ZAL<br>β-ZAL<br>β-ZEL<br>ZAN<br>TeA<br>AOH<br>AME<br>DON<br>DOM-1<br>3/15-AcDON<br>T-2<br>HT-2<br>T2-G<br>AFB_1<br>AFM_1<br>OTA<br>ENNA<br>$ENNA_1$<br>ENNB<br>$ENNB_1$<br>BEA	-	5<br>1<br>5<br>1<br>1<br>1<br>1<br>5<br>1<br>5<br>5<br>1<br>1<br>5<br>5<br>1<br>1<br>1<br>1<br>1<br>1<br>1<br>1	41.2 - 266.1 (R_E)	35.4<br>22.4<br>18.5<br>30.5<br>40.3<br>30.3<br>12.4<br>13.5<br>22.7<br>27.5<br>23.7<br>16.7<br>82.4<br>62.8<br>66.4<br>35.0<br>35.3<br>68.1<br>78.6<br>304.5<br>61.9<br>72.8<br>64.3	Waters Acquity HSS T3 column (100 mm × 2.1 mm i.d., dp: 1.8 μm)	10 mM ammonium formate, 0.3% formic acid in water and 10 mM ammonium formate, 0.3% formic acid in methanol	Agilent 1290 series UHPLC system coupled to a Sciex 6500 QTrap mass spectrometer	[9]<br>[10]
serum	SPE columns<br>Oasis™ HLB (Waters)	-	β-glucuronidase type H-2 from Helix pomatia	ZEN<br>α-ZEL<br>β-ZEL<br>ZAN<br>α-ZAL<br>β-ZAL<br>DON<br>DOM-1	0.03<br>0.24<br>0.71<br>0.13<br>0.16<br>0.28<br>0.14<br>0.23	0.08<br>0.78<br>2.37<br>0.43<br>0.52<br>0.94<br>0.45<br>0.76	85-117		100× 2 mm Pursuit XRs Ultra 2.8 μm C18 column (Agilent Tech-nologies)	water and MeOH/ACN (70/30 v/v)	1200 series HPLC system (Agilent Technologies) coupled to a 4000 QTrap (Applied Biosystems)	[2]<br>[13]

Matrix	Sample preparation	Creatinine adjustment	Enzymatic hydrolysis (enzyme)	Analytes	LOD [ng/mL]	LOQ [ng/mL]	Recovery [%]	Matrix effect (SSE) [%]	Analytical column	Mobile phase	LC-MS	Reference
		-	-	T-2<br>HT-2<br>T-2 triol	0.3<br>0.6<br>2	1<br>2<br>5	74.3- 102.4	79.3-93.8	Zorbax XDB-C18 column (150 mm × 2.1 mm, 3.5 µm; Agilent, USA)	5.0 mM ammonium acetate in water and acetonitrile	1290 Infinity series UHPLC system (Agilent Technologies, Waldbronn, Germany) coupled to a 4000 QTrap mass spectrometer	[8]
serum	LLE<br>ACN	-	-	DON<br>DOM-1<br>ZEN<br>ZAN<br>α- ZEL<br>β-ZEL<br>α-ZAL<br>β-ZAL<br>OTA<br>T-2<br>HT-2<br>FB_1<br>AFB_1	0.40<br>0.22<br>0.52<br>0.05<br>0.02<br>0.02<br>0.02<br>0.02<br>0.02<br>0.05<br>0.15<br>0.01<br>0.05	10<br>5<br>5<br>5<br>5<br>5<br>5<br>5<br>5<br>2<br>5<br>1<br>2	77.57(OTA) - 109.8 (ZAN)	85.5<br>95.4<br>90.7<br>90.6<br>83.7<br>83.7<br>87,7<br>87.7<br>87.9<br>108.7<br>80.2<br>98.8<br>98.7	Hypersil Gold column (50 mm × 2.1 mm i.d., dp: 1.9 µm) ThermoFisher Scientific	0.1% acetic acid in water and methanol	LC ThermoFisher Scientific (Breda, The Netherlands) TSQ® Quantum Ultra triple quadrupole mass spectrometer ThermoFisher Scientific	[14]
		-	β-Glucuronidase Type HP-2 from Helix pomatia	ZEN<br>α-ZEL<br>β-ZEL<br>α-ZAL<br>β-ZAL<br>ZAN	0.003<br>0.012<br>0.020<br>0.020<br>0.020<br>0.004<br>0.040	0.2-5	83.5 - 86.0 (R_E)<br>82.4-96.1 (R_A)	97–112	Hypersil Gold column (50 mm × 2.1 mm i.d., dp: 1.9 µm) ThermoFisher Scientific	0.01% acetic acid in water, and acetonitrile	LC ThermoFisher Scientific (Breda, The Netherlands) TSQ® Quantum Ultra triple quadrupole mass spectrometer ThermoFisher Scientific	[15]

Matrix	Sample preparation	Creatinine adjustment	Enzymatic hydrolysis (enzyme)	Analytes	LOD [ng/mL]	LOQ [ng/mL]	Recovery [%]	Matrix effect (SSE) [%]	Analytical column	Mobile phase	LC-MS	Reference
serum	LLE<br>ACN	-	-	BEA<br>ENNA<br>$ENNA_1$<br>ENNB<br>$ENNB_1$	0.010<br>0.001<br>0.003<br>0.004<br>0.008	0.2<br>0.1<br>0.1<br>0.2<br>0.2	81.94 (BEA) – 94.29 (ENNB)<br><br>(R_E)	90.6<br>111<br>81.5<br>91.6<br>92.3	Hypersil Gold column (50 mm × 2.1 mm i.d., dp: 1.9 µm)	0.1% glacial acetic acid in water and ACN	LC TSQ® Quantum Ultra triple quadrupole mass spectrometer ThermoFisher Scientific	[16]
		-	-	DON<br>DOM-1<br>3-AcDON<br>15-AcDON	0.01<br>0.07<br>0.01<br>0.2	0.1<br>0.5<br>1.0<br>1.0	28.2 (15-AcDON)-88.3 (DON)	81.5<br>97.0<br>80.3<br>44.4	Hyper-sil Gold (50 × 2.1 mm i.d., 1.9 µm) column	water +0.1% glacial acetic acid and methanol +0.1% glacial acetic acid	Acquity UPLC system Xevo® TQ-S mass spectrome-ter (Waters, Zellik, Belgium)	[17]<br><br>[18]
	LLE<br>ACN<br>with 0.1% formic acid	-	-	ZEN<br>α-ZEL<br>α-ZAL<br>β-ZAL<br>β-ZEL<br>ZAN<br>TeA<br>AOH<br>AME<br>DON<br>DOM-1<br>3/15-<br>AcDON<br>T-2<br>HT-2<br>T2-G<br>AFB_1<br>AFM_1<br>OTA<br>ENNA<br>$ENNA_1$<br>ENNB<br>$ENNB_1$<br>BEA	0.001 - 1.68	1<br>1<br>1<br>1<br>1<br>1<br>1<br>1<br>1<br>1<br>1<br>1<br>1<br>1<br>1<br>2<br>1<br>1<br>1<br>1<br>1<br>1<br>1<br>1	72.9 (TEA)-132.2 (ENNA)<br>(R_E)	86.2<br>74.7<br>73.0<br>74.4<br>79.1<br>86.5<br>96.3<br>114.9<br>90.1<br>40.4<br>42.6<br>93.5<br>83.3<br>61.7<br>71.5<br>87.4<br>50.1<br>101.3<br>62.1<br>66.8<br>50.9<br>45.5<br>64.3	Waters Acquity HSS T3 column (100 mm × 2.1 mm i.d., dp: 1.8 µm)	10 mM ammonium formate, 0.3% formic acid in water and 10 mM ammonium formate, 0.3% formic acid in methanol	Agilent 1290 series UHPLC system coupled to a Sciex 6500 QTrap mass spectrometer	[9]<br>[10]

References

1. Jodlbauer, J.; Zöllner, P.; Lindner, W. Determination of zeranol, taleranol, zearalenone, α- and β-zearalenol in urine and tissue by high-performance liquid chromatography-tandem mass spectrometry. *Chromatographia* **2000**, *51*, 681–687, doi:10.1007/BF02505405.
2. Brezina, U.; Rempe, I.; Kersten, S.; Valenta, H.; Humpf, H.U.; Dänicke, S. Diagnosis of intoxications of piglets fed with Fusarium toxin-contaminated maize by the analysis of mycotoxin residues in serum, liquor and urine with LC-MS/MS. *Arch. Anim. Nutr.* **2014**, *68*, 425–447, doi:10.1080/1745039X.2014.973227.
3. Gambacorta, S.; Solfrizzo, H.; Visconti, A.; Powers, S.; Cossalter, A.M.; Pinton, P.; Oswald, I.P. Validation study on urinary biomarkers of exposure for aflatoxin B_1, ochratoxin A, fumonisin B_1, deoxynivalenol and zearalenone in piglets. *World Mycotoxin J.* **2013**, *6*, 299–308, doi:10.3920/WMJ2013.1549.
4. Gambacorta, L.; Olsen, M.; Solfrizzo, M. Pig urinary concentration of mycotoxins and metabolites reflects regional differences, mycotoxin intake and feed contaminations. *Toxins (Basel).* **2019**, *11*, 378, doi:10.3390/toxins11070378.
5. Nagl, V.; Woechtl, B.; Schwartz-Zimmermann, H.E.; Hennig-Pauka, I.; Moll, W.D.; Adam, G.; Berthiller, F. Metabolism of the masked mycotoxin deoxynivalenol-3-glucoside in pigs. *Toxicol. Lett.* **2014**, *229*, 190–197, doi:10.1016/j.toxlet.2014.06.032.
6. Thanner, S.; Czeglédi, L.; Schwartz-Zimmermann, H.E.; Berthiller, F.; Gutzwiller, A. Urinary deoxynivalenol (DON) and zearalenone (ZEA) as biomarkers of DON and ZEA exposure of pigs. *Mycotoxin Res.* **2016**, *32*, 69–75, doi:10.1007/s12550-016-0241-2.
7. Binder, S.B.; Schwartz-Zimmermann, H.E.; Varga, E.; Bichl, G.; Michlmayr, H.; Adam, G.; Berthiller, F. Metabolism of zearalenone and its major modified forms in pigs. *Toxins (Basel).* **2017**, *9*, 56, doi:10.3390/toxins9020056.
8. Sun, Y.; Zhang, G.; Zhao, H.; Zheng, J.; Hu, F.; Fang, B. Liquid chromatography-tandem mass spectrometry method for toxicokinetics, tissue distribution, and excretion studies of T-2 toxin and its major metabolites in pigs. *J. Chromatogr. B Anal. Technol. Biomed. Life Sci.* **2014**, *958*, 75–82, doi:10.1016/j.jchromb.2014.03.010.
9. Lauwers, M.; De Baere, S.; Letor, B.; Rychlik, M.; Croubels, S.; Devreese, M. Multi LC-MS/MS and LC-HRMS methods for determination of 24 mycotoxins including major phase I and II biomarker metabolites in biological matrices from pigs and broiler chickens. *Toxins (Basel).* **2019**, *11*, 171, doi:10.3390/toxins11030171.
10. Lauwers, M.; Croubels, S.; Letor, B.; Gougoulias, C.; Devreese, M. Biomarkers for Exposure as A Tool for Efficacy Testing of A Mycotoxin Detoxifier in Broiler Chickens and Pigs. *Toxins (Basel).* **2019**, *11*, 187, doi:10.3390/toxins11040187.
11. Rui Guo, W.; Xiao Ou, S. Simultaneous Detection Method for Mycotoxins and their Metabolites in Animal Urine by Using Impurity Adsorption Purification followed by Liquid Chromatography-Tandem Mass Detection. *J. Chromatogr. Sep. Tech.* **2015**, *6*, 308, doi:10.4172/2157-7064.1000308.
12. Song, S.; Ediage, E.N.; Wu, A.; De Saeger, S. Development and application of salting-out assisted liquid/liquid extraction for multi-mycotoxin biomarkers analysis in pig urine with high performance liquid chromatography/tandem mass spectrometry. *J. Chromatogr. A* **2013**, *1292*, 111–120, doi:10.1016/j.chroma.2012.10.071.
13. Brezina, U.; Valenta, H.; Rempe, I.; Kersten, S.; Humpf, H.U.; Dänicke, S. Development of a liquid chromatography tandem mass spectrometry method for the simultaneous determination of zearalenone, deoxynivalenol and their metabolites in pig serum. *Mycotoxin Res.* **2014**, *30*, 171–186, doi:10.1007/s12550-

014-0200-8.
14. Devreese, M.; De Baere, S.; De Backer, P.; Croubels, S. Quantitative determination of several toxicological important mycotoxins in pig plasma using multi-mycotoxin and analyte-specific high performance liquid chromatography–tandem mass spectrometric methods. *J. Chromatogr. A* **2012**, *1257*, 74–80, doi:10.1016/j.chroma.2012.08.008.
15. Acta, A.C.; De Baere, S.; Osselaere, A.; Devreese, M.; Vanhaecke, L.; De Backer, P.; Croubels, S. Development of a liquid–chromatography tandem mass spectrometry and ultra-high-performance liquid chromatography high-resolution mass spectrometry method for the quantitative determination of zearalenone and its major metabolites in chicken and pig plasma. *Anal. Chim. Acta* **2012**, *756*, 37–48, doi:10.1016/j.aca.2012.10.027.
16. Devreese, M.; De Baere, S.; De Backer, P.; Croubels, S. Quantitative determination of the Fusarium mycotoxins beauvericin, enniatin A, A1, B and B1 in pig plasma using high performance liquid chromatography–tandem mass spectrometry. *Talanta* **2013**, *106*, 212–219, doi:10.1016/j.talanta.2012.11.068.
17. Broekaert, N.; Devreese, M.; De Mil, T.; Fraeyman, S.; De Baere, S.; De Saeger, S.; De Backer, P.; Croubels, S. Development and validation of an LC–MS/MS method for the toxicokinetic study of deoxynivalenol and its acetylated derivatives in chicken and pig plasma. *J. Chromatogr. B* **2014**, *971*, 43–51, doi:10.1016/j.jchromb.2014.09.012.
18. Broekaert, N.; Devreese, M.; De Mil, T.; Fraeyman, S.; Antonissen, G.; De Baere, S.; De Backer, P.; Vermeulen, A.; Croubels, S. Oral Bioavailability, Hydrolysis, and Comparative Toxicokinetics of 3-Acetyldeoxynivalenol and 15-Acetyldeoxynivalenol in Broiler Chickens and Pigs. *J. Agric. Food Chem.* **2015**, *63*, 8734–8742, doi:10.1021/acs.jafc.5b03270.
